# Alternative Antigen Processing for MHC Class I: Multiple Roads Lead to Rome

**DOI:** 10.3389/fimmu.2015.00298

**Published:** 2015-06-05

**Authors:** Cláudia C. Oliveira, Thorbald van Hall

**Affiliations:** ^1^Department of Clinical Oncology, Leiden University Medical Center, Leiden, Netherlands

**Keywords:** transporter associated with antigen processing, peptide loading complex, ER-associated degradation, autophagy, CD8 T cells, T cell epitopes associated with peptide processing, tumor immunology

## Abstract

The well described conventional antigen-processing pathway is accountable for most peptides that end up in MHC class I molecules at the cell surface. These peptides experienced liberation by the proteasome and transport by the peptide transporter TAP. However, there are multiple roads that lead to Rome, illustrated by the increasing number of alternative processing pathways that have been reported during last years. Interestingly, TAP-deficient individuals do not succumb to viral infections, suggesting that CD8 T cell immunity is sufficiently supported by alternative TAP-independent processing pathways. To date, a diversity of viral and endogenous TAP-independent peptides have been identified in the grooves of different MHC class I alleles. Some of these peptides are not displayed by normal TAP-positive cells and we therefore called them TEIPP, for “T-cell epitopes associated with impaired peptide processing.” TEIPPs are hidden self-antigens, are derived from normal housekeeping proteins, and are processed via unconventional processing pathways. Per definition, TEIPPs are presented via TAP-independent pathways, but recent data suggest that part of this repertoire still depend on proteasome and metalloprotease activity. An exception is the C-terminal peptide of the endoplasmic reticulum (ER)-membrane-spanning ceramide synthase Trh4 that is surprisingly liberated by the signal peptide peptidase (SPP), the proteolytic enzyme involved in cleaving leader sequences. The intramembrane cleaving SPP is thereby an important contributor of TAP-independent peptides. Its family members, like the Alzheimer’s related presenilins, might contribute as well, according to our preliminary data. Finally, alternative peptide routing is an emerging field and includes processes like the unfolded protein response, the ER-associated degradation, and autophagy-associated vesicular pathways. These data convince us that there is a world to be discovered in the field of unconventional antigen processing.

## Classical Antigen Presentation Pathway Represents Only One Side of the Story

Antigen-specific CD8 T-cells recognize peptides of 8–10 amino acids long that are associated with MHC class I/β_2_m complexes. Cell surface expression of MHC class I/β_2_m/peptide complexes is the end result of a process that begins inside the cell where proteolysis of aged, misfolded, or defective ribosomal product (DRiP) proteins generates small peptides. Potentially, a multitude of proteolytic systems may generate antigenic peptides, but the proteasome is responsible for the liberation of majority of them. Inhibition of proteasome activity strongly decreased the pool of MHC class I-binding peptides (1). Proteasomal cleavage typically creates a peptide’s C-terminus compatible with MHC class I binding, and peptides are typically extended at their N-terminus (2, 3). Peptides generated in the cytosol are translocated into the endoplasmic reticulum (ER) by the TAP1/TAP2 peptide transporter, where they have access to the peptide loading complex (PLC), which is located within the ER. TAP is a heterodimeric member of the ATP-binding cassette (ABC) family of transporters, and peptide binding induces ATP hydrolysis and transport across the ER membrane. Once in contact with the PLC, the ER-amino peptidase ERAAP (also known as ERAP1) trims N-extended peptides to a length appropriate for MHC class I binding (4–6). Chaperone tapasin promotes the formation of stable MHC class I/peptide complexes and acts as an editor. Additionally, calnexin facilitates the early folding of MHC class I heavy chains, whereas calreticulin and ERp57 are involved in peptide loading (7–9). This pathway is also known as the proteasome-TAP pathway and is considered as the conventional processing route because it is the mainstream pathway operating in cells under normal conditions (10–12). However, cells are equipped with alternative routes leading to liberation and loading of peptides into MHC class I molecules. These routes are independent of one or more molecules from the conventional pathway such as the proteasome, tapasin, or TAP. This has become apparent from studies on cells with deficiencies in the conventional processing pathway. In this review, we will discuss what is known to date regarding alternative enzymes and routes to peptide loading compartments of endogenously generated peptides that feed the direct MHC class I pathway, especially important in cases of failure of the conventional route. We have not included interesting literature on cross-presentation pathways for MHC class I peptides.

## Proteasome-Independent Pathways: Enzymes Replacing Proteasomal Activity for Liberation of Peptides

More than 10 years ago, several papers made the important discovery that a large oligopeptidase, called tripeptidyl peptidase II (TPPII), participates in endoproteolytic activity in the cytosol and partially compensates for a deficient proteasomal activity (13–16). Increased TPPII activity even allowed for cell survival in lethal conditions of proteasome inhibition (14, 16). In these conditions, TPPII activity also partially restored peptide presentation in MHC class I molecules and it was speculated that it could account for the generation of some epitopes independently or in cooperation with the proteasome (16). In fact, a paper from Seifert et al. showed that TPPII was involved in the generation of an epitope from the human immunodeficiency virus (HIV) protein negative factor (Nef) (17). After that, an increasing number of proteolytic enzymes have been implicated in the generation of peptide-epitopes independently of the proteasome. Insulin-degrading enzyme (IDE) generates an epitope from the human melanoma antigen MAGE-A3 (18). Thimet oligopeptidase (TOP) and nardilysin are required for the generation of three other clinically relevant CTL epitopes: the tumor-antigen PRAME, an epitope from Epstein–Barr virus (EBV) protein EBNA3C, and an epitope from the melanoma protein MART-1 (15). These enzymes are part of an array of cytosolic endo- and exo-proteases that complement proteasomal activity and degrade proteasome products ultimately into amino acids. Importantly, the process of peptide liberation from the protein context is inevitably coupled to the destruction pathway and all proteases mentioned above also destroy some antigenic peptides (19–22). Peptides that are “rescued” from total destruction are transported by TAP into the ER and can potentially bind MHC class I molecules.

### Leader sequences are liberated by signal peptidase and signal peptide peptidase

In eukaryotic cells, secretory and membrane proteins contain a signal sequence essential for protein targeting to the ER, the entrance for the secretory pathway (23, 24). These signal sequences are typically composed of three domains: a hydrophobic core (h region) of 6–15 amino acids, a polar C-terminal end (c region) with small uncharged amino acids, and a polar N-terminal region (n region) with a positive net charge (25). After insertion into the protein-conduction channel, signal peptides are usually cleaved from the preprotein by signal peptidase (SP) (26). Thereafter, signal peptides, which are small domains and trapped in the ER membrane, can undergo intramembrane proteolysis by cleavage within their transmembrane region by the presenilin-type aspartic protease signal peptide peptidase (SPP) (25, 27). Peptide ligands suitable for MHC class I binding are thought to be generated after the intramembrane proteolysis by SPP that promotes the release of signal peptide fragments from the ER membrane (28, 29). The SPP-cleaved fragments in the vicinity of the cytosol can get access to the cytosol again and be further processed by the proteasome and transported by TAP into the ER. Most HLA class I molecules donate their leader sequences for binding to the non-classical HLA-E, and the cleavage of these signal sequences is mediated by SP and SPP (30–33). These leader peptides are even the most dominant source of peptides for HLA-E. Proper surface expression of HLA-E prevents cytotoxic action of natural killer cells that continuously sense the presence of peptide/HLA-E complexes through their CD94/NKG2 receptors (34–36). The absence of these complexes at cell surface results in failure of interaction with CD94/NKG2A receptors, which activate NK cells for killing their targets.

**Figure 1:**
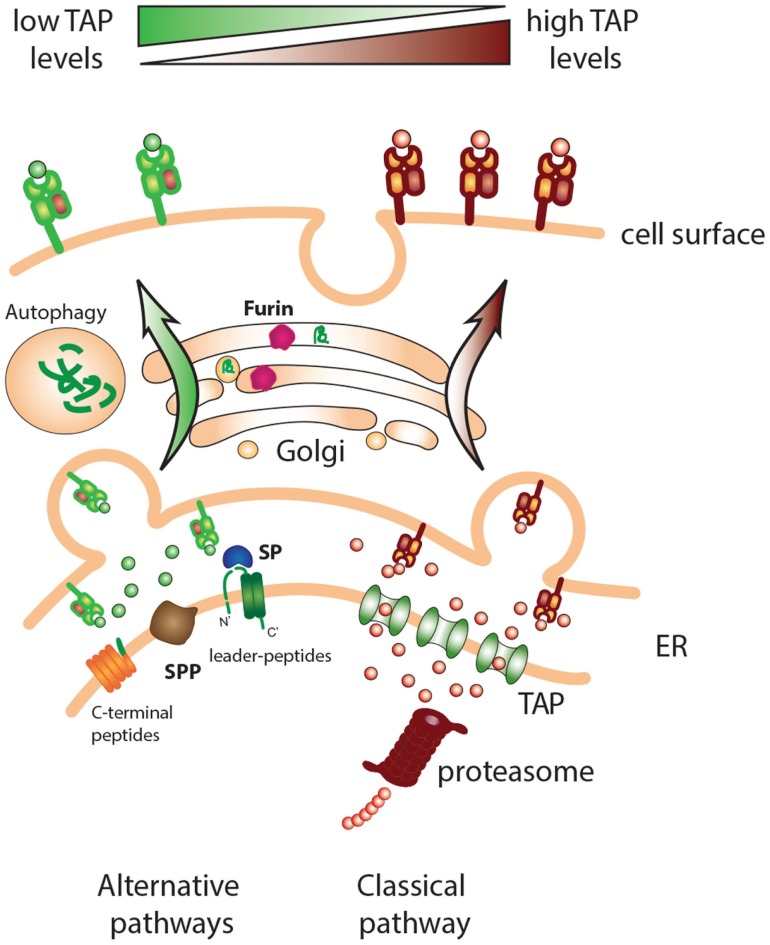
**Classical and alternative pathways for MHC class I presentation**. Cells with deficiencies in components of the MHC class I processing pathway, such as TAP, can present a repertoire of peptides derived from alternative processing pathways. Different “housekeeping” cell functions such as signal peptide cleavage, protein maturation in the Golgi, and protein/organelle disposal via autophagy can provide peptide ligands for MHC class I loading.

Pioneering studies by Peter Cresswell and Victor Engelhard in 1992 revealed that most peptides presented at the surface of TAP-deficient cells were derived from signal sequences, specific protein regions at the N-terminus of proteins (37, 38). Indeed, the parts of the leader peptide within the ER membrane that are closest to the ER lumen are released there and can get access to MHC class I grooves independent of proteasomes or TAP (Figure 1). After the cleavage of the transmembrane sequence by SPP, the peptide fragments are released and can associate with MHC class I/β_2_m nascent molecules. This intramembrane proteolysis by SPP is thought to be important for the clearance of the ER membrane by removing small protein remnants anchored at the membrane rendering them susceptible for subsequent degradation. The intramembrane cleavage by SPP is favored by SPP topology that conceals the catalytic center within the plane of the membrane. The two aspartic residues required for the proteolytic activity of SPP are located within conserved (Y/F)D and G(L/I/F)GD amino acid motifs in two adjacent transmembrane domains (39, 40). Regarding a cleavage motif for SPP, no consensus cleavage site has been described. However, SPP demonstrates a strong preference for substrates with helix-destabilizing residues in their transmembrane domain (41–43). Amino acids like asparagine, serine, and cysteine disturb a perfect alfa-helical conformation of the transmembrane domain and are therefore referred to as “helix-bending” or “helix-breaking” residues. Signal peptides have been shown to contain amino acids with “helix-breaking” capacity within their h region, which critically influence their proteolytic processing by SPP (42, 44–46). The disturbance of the α-helix caused by these residues is thought to facilitate intramembrane proteolysis. This and other issues would be clarified with the atomic resolution of this protease but this is still lacking due to its technical difficulties. We can have an approximation of that by looking at the crystal structure of presenilin/SPP homologs recently published (47–50). JR1 is an SPP homolog from the archaeon *Methanoculleus marisnigri* that harbors nine transmembrane helices, similar to what is predicted for SPP, with TMD 6 and TMD 7 containing the YD and GxGD motif, respectively. The two catalytic aspartate residues are located close to each other and approximately 8 Å into the lipid membrane. Proteolytic activity occurs in the presence of water molecules that gain access to the catalytic aspartates through a large cavity between two terminal domains (48). The three-dimensional structure of a human presenilin comprised into the γ-secretase complex has also been described (51). For the near future, we can expect more information on the catalytic activity of this family of proteases, including SPP, which is definitely an important contributor of peptides for MHC class I presentation.

### Additional functions for the signal peptide peptidase

Our recent data revealed an additional role for intramembrane proteolysis by SPP. Regardless its name, SPP also appeared to liberate a C-terminal peptide, independent of proteasome activity. The processing of C-terminal regions of a type II protein inserted in the ER membrane leads to the presentation of peptides independently of proteasome and TAP (52, 53). The C-terminal region from the ceramide synthase Trh4, which is a multiple membrane-spanning protein in the ER, contains a 9-mer peptide-epitope that is located at the very C-terminal end of the protein and protrudes into the lumen of the ER (53–56). The Trh4 protein has a housekeeping function and is ubiquitously expressed. Inhibition of SPP activity blocked the generation of the Trh4 peptide. Experiments with mutant forms of the Trh4 protein indicated that the intramembrane cleavage by SPP occurs at the direct upstream region of the T cell epitope within the lipid bilayer (52). We speculated that SPP activity in the ER membrane is sufficient to liberate the minimal C-terminal 9-mer peptide and release of this peptide into the ER lumen. Other proteolytic enzymes, such as amino-peptidases, were dispensable. Further carboxy-terminal processing was not needed since the epitope is located at the very C-terminal end of the protein. Direct release of the liberated peptide into the ER lumen is very likely, due to the type II transmembrane orientation of the Trh4 protein tail (N-terminus to C-terminus orientation). The exact peptide loading mechanism of the Trh4 membrane peptide, however, remains to be determined.

What triggers the cleavage of Trh4 by SPP is not known yet. In addition to its liberation function of small transmembrane substrates, SPP has been shown to associate with misfolded membrane proteins in complexes where SPP is represented as a monomer, dimer, or multimer (57). It was suggested that such high molecular weight complexes act as chaperones to dispose of membrane aggregates (57, 58). The role of SPP in this degradation machinery might be to liberate such aggregates from the ER membrane. These discoveries were based on the viral US2 and US11 proteins, which successfully labels MHC class I molecules in the ER for retrograde transport to push this protein back to the cytosol for degradation by proteasomes (59, 60). Interestingly, the SPP family member, presenilin 2, seems to be associated with this membrane proteolysis as well (61). Since SPP and presenilin 2 have opposing preference for type I and type II transmembrane orientations, such a “degradome” machinery might be responsible to clear the ER membranes. This type of machinery is called the ER-associated degradation (ERAD) pathway. ERAD is an ER quality control system, which monitors the integrity of nascent or ER-resident proteins and targets incorrectly folded or misassembled proteins to degradation. The disposal of misfolded MHC class I heavy chains also occurs through the ERAD pathway in the absence of viral interference (62). Clearly, viruses take advantage of these existing pathways and hijack them in order to evade immune recognition by cytotoxic T cells (63, 64).

Another role for SPP in ERAD has come from a recent paper describing the cleavage of the unfolded protein response (UPR) regulator XBP1u by SPP (65). XBP1u is a type II membrane protein and undergoes intramembrane cleavage within a conserved type II TM domain while integrated in a complex containing SPP, the protease Derlin-1, and the E3 ligase TRC8, which prime SPP for XBP1u cleavage. An ectodomain shedding of SPP substrates prior to SPP cleavage is thought to be required and normally performed by SP in signal sequences but such cleavage was unnecessary in the case of XBP1u, similarly to what we have described for Trh4 (52, 65). In general, the UPR induces a strong downregulation of MHC class I molecules at the cell surface (66, 67).

Thus, SPP activity seems to have a direct impact on MHC class I peptide presentation by cleavage of leader sequences from nascent proteins and the liberation of some C-termini for MHC class I loading. A more indirect role that impacts on MHC class I presentation has been revealed and occurs through an ERAD pathway. This is also supported by a recent study using a systems-level strategy reporting the involvement of SPP in the network that coordinates the ERAD response (68).

### Liberation of peptides in the secretory route: Furin

The group of Yewdell showed more than a decade ago that peptides located at the C-terminus of ER-targeted proteins are very efficiently generated and presented on MHC class I (69–72). The location of the peptide was essential to be at the very end of the C-terminus of the protein, not requiring C-terminal trimming, in line with the fact that there is poor carboxypeptidase activity in the ER (73). They described the presentation of TAP-independent peptides from one ER-resident protein, Jaw1, and proteins in the secretory pathway, like ovalbumin and CD23. In each case, the peptides were efficiently liberated from the very C-terminus by the activity of yet unidentified endo-proteases to be generated as class I ligands. Based on this pathway of peptide liberation, the authors provided the term “C-end rule” to highlight the capacity of ER-resident proteases to liberate class I ligands from the C-terminal ends of ER-targeted proteins. The liberation of peptides without the intervention of the proteasome can also occur in the trans-Golgi network (74, 75). The main proteolytic enzyme involved was shown to be furin, a known protease of the trans-Golgi network normally required for the maturation of secreted proteins (e.g., growth factors and neurotransmitters) by cleaving at precise stretches of three to four basic residues (76). Furin is part of a family of proprotein convertases that comprises nine members (77). Three members (PC5/6, PACE4, and PC7) including furin are widely expressed and together they take part in a variety of processes occurring in the trans-Golgi network, cell surface, or endosomes. This leads to the activation or inactivation of receptors, ligands, enzymes, viral glycoproteins, or growth factors (78). Furin also has important functions during development by processing substrates like bone morphogenetic protein 10 (BMP10), a member of the TGF-β superfamily that plays a critical role in heart development (79). Furin processes a wide variety of precursor proteins after the C-terminal arginine residue in the preferred consensus motif –Arg–X–Arg/Lys–Arg ↓–X– (X is any amino acid and “ ↓” indicates the cleavage position) (80). Initially, this pathway was studied with the use of a model peptide at the C-terminus of the secreted Hepatitis HBe protein. Furin-processed antigens targeted to the secretory route were presented by MHC class I at the cell surface and could elicit functional CD8 T-cell responses *in vivo* in a TAP-independent fashion (75, 81). The C-termini of secretory or ER-localized proteins thus appear to be processed for presentation to CD8 T cells (Figure 1).

## TAP-Independent Pathways: Alternative Routing to MHC Class I Loading Compartments

The generation of MHC class I ligands described above defines several alternative ways to generate peptide ligands without the intervention of the proteasome. These represent unusual pathways for peptide generation. Now, we will discuss a different constraint in the conventional antigen presentation pathway related to the blockade of peptide entrance in the ER due to TAP deficiency. In human beings, TAP-deficiency syndrome has been described in several independent families and results from mutations in either one of the subunits of the peptide transporter, TAP1 and TAP2 (82, 83). Interestingly, these TAP-deficient individuals do not succumb to viral infections, suggesting that CD8 T cell immunity is sufficiently supported by alternative TAP-independent processing pathways. To date, a diversity of viral and endogenous TAP-independent peptides have been identified in the grooves of different MCH class I alleles. Importantly, these TAP-deficient patients harbor a polyclonal CD8 T-cell repertoire that is capable of recognizing peptides from the EBV virus, like protein LMP2, presented on TAP-deficient cells (84). The TAP-independent processing pathway is capable of generating enough MHC class I/peptide complexes in order to keep immunosurveillance and control of viral infections. Studies with TAP1-knockout mice have shown that surface expression levels of MHC class I are indeed lower, but the remaining complexes do induce a broad and polyclonal repertoire of CD8 T-cells (85–87). The TCR usage was shown to be very comparable to that of wild-type mice and TAP1-knockout mice were capable of mounting anti-viral CD8 T cell responses. Together, these data show that, although crippled, the MHC class I-presented peptide repertoire in the absence of TAP is sufficient to support CD8 T cell immunity.

### TAP-independent t cell epitopes: TEIPP

Interestingly, peptides emerging from alternative TAP-independent routes appeared to be immunogenic. Following immunizations in mice with TAP-deficient tumor cells, specific CD8 T-cells were induced that recognize TAP-deficient cells, but not normal cells (56, 88). These T-cell epitopes seemed to be selectively presented by TAP-deficient cells but not under normal conditions. This alternative peptide repertoire emerges due to processing defects and therefore these peptides were named “*T cell epitopes associated with impaired peptide processing*” (TEIPP). The molecular identification of some TEIPP peptides revealed that they can be diverse in length (from 9-mer to 18-mer), amino acid composition, and MHC class I binding, as some are presented by classical MHC class I molecules and others by the non-classical MHC molecule HLA-E and the mouse homolog Qa-1^b^ (Table 1) (35, 89–92). They are derived from normal housekeeping proteins with ubiquitous expression, but are surprisingly not loaded on MHC class I in cells with an intact antigen-processing machinery. They constitute normal self-peptides (non-mutated, not pathogen- or tumor-specific) and can be regarded as real neo-antigens. The immunogenicity of TEIPP peptides exists because they are not presented by normal cells including the thymus. During thymic development, T cells are subjected to two subsequent processes called positive and negative selection (93, 94). Negative selection is necessary for the maintenance of self-tolerance as it induces the deletion or inactivation of potentially autoreactive thymocytes (95). We recently demonstrated that TEIPP-specific CD8 T-cells indeed do not undergo negative selection and are thereby available for therapeutic exploitation. Since the peptides recognized by TEIPP-specific CTL are derived from housekeeping proteins, we tried to understand why TEIPPs are not presented by processing intact cells.

**Table 1:** **TEIPP peptide-epitopes defined thus far based on CD8 T-cell recognition**.

Peptide sequence/MHC class I	Source protein	Location in protein	Responsible enzyme	Presented upon	Reference
MCLRMTAVM H2-D^b^	Trh4 Q9D6K9 (1)	C-terminal	SPP (2)	TAP deficiency	(53)
“mi3 epitope” H2-K^b^	Unknown	Unknown	Proteasome	TAP deficiency	(119)
FAPLPRLPTL Qa-1^b^ (3)	Acyl carrier protein Q9CR21	N-terminal	Unknown	TAP deficiency	(91)
FYAEATPML Qa-1^b^	Fam49b “hypothetical protein” NM_144846	Central	Unknown	ERAAP (4) deficiency	(92)
VLLQAGSLHA HLA-A2*0201	Preprocalcitonin P01258	Signal sequence	SP (5) and SPP	TAP deficiency	(97)

*(1) UniProtKB database*.

*(2) SPP is signal peptide peptidase*.

*(3) Qa-1^b^ is the mouse homolog of HLA-E*.

*(4) ERAAP is ER-amino-peptidase*.

*(5) SP is signal peptidase*.

Collectively, our data show that TEIPP peptides are actually produced within processing proficient cells, but somehow are not or not sufficiently presented by their surface MHC class I molecules. Taking the Trh4-derived TEIPP peptide as a model, we have analyzed the expression of the *Trh4* gene in several epithelial populations isolated from wild-type and TAP1-ko mice (53). This analysis revealed the same level of RNA transcripts between the normal and knockout populations, suggesting comparable protein levels in both cell types. The liberation of the Trh4 peptide is performed by SPP, which is active in TAP-positive as well as TAP-negative targets (52). Overexpression of the Trh4 gene in TAP-positive cells leads to surface presentation in MHC class I (53), still in a proteasome-independent way. Moreover, proteasome inhibition in TAP-positive cells results in presentation of the endogenous Trh4 peptide (56), indicating that, indeed, this TEIPP peptide is generated in all cells but loses competition with the overwhelming amount of TAP-imported peptides in TAP-positive cells. Some alternative peptides, like the ones derived from EBV proteins were shown to be presented on TAP-positive cells to comparable extent, although using alternative pathways. Moreover, our data suggest that the limited quantity of the Trh4 peptide-epitope in the ER is the main cause of selective presentation in TAP-deficient cells. Interestingly, gradual increase of overexpression correlated with the degree of recognition by the TEIPP-specific CTL clone, implying that TAP transport actually constitutes a strong barrier for TEIPP peptides. The study of human TEIPP antigens corroborates these findings. One antigenic peptide is encoded by the human *CALCA* gene and derives from the signal sequence of preprocalcitonin (ppCT) protein. This peptide is liberated in the ER lumen by sequential cleavage with SP and SPP, independently from proteasomes (Table 1) (96). The presentation of the ppCT peptide to specific CTL was found in human lung and medullary thyroid carcinomas that had very low expression of TAP. Presentation of the ppCT peptide occurred also in normal non-transformed cells, such as dendritic cells (DCs), after knockdown of TAP. Overexpression of the *CALCA* gene in DCs and TAP-positive tumor cells resulted in recognition by the specific CTL clone (97). Identification of additional human TEIPP antigens at the molecular level will enable CD8 T cell targeting of otherwise CTL-resistance TAP-negative tumor variants (98, 99). Together, these findings support the model of peptide competition in the ER as a factor that prevents presentation of peptides from alternative sources, and shape a picture of alternative processing pathways that emerge upon defects in the conventional one (Figure 1).

### How to reach peptide loading complexes when you bypass TAP?

The precise loading mechanism of TAP-independent signal peptides into MHC class I molecules is not known, since processing by SP and SPP is thought to take place outside of the PLC. This sophisticated machinery for optimizing ligand length and quality and facilitating peptide loading onto nascent MHC class I molecules greatly facilitates peptide loading by physical bridging transporters to chaperones for loading and also “edits” the repertoire of bound peptides to maximize their affinity (7). The PLC molecule tapasin tethers MHC class I molecules to the peptide transporter acting together with the chaperone calreticulin and the oxidoreductase ERp57 (100). Tapasin can sense the quality of peptide bound by MHC class I complexes, and allows successive rounds of peptide binding until a certain affinity threshold is met. Trimming of incoming peptides by ERAAP can be necessary for obtaining a good peptide length before selection by a defined MHC class I allele. A recent paper states that MHC class I molecules initially bind a variety of peptides, including some low-affinity or N-terminally extended ones but then quickly dissociate from the molecule followed by the selection of the best fitting candidates (101). However, TAP-independent leader peptides do not arrive in the ER via TAP and thus lack these editing and optimizing chaperones. In the absence of TAP transporters, the loading of peptides into MHC class I occurs without a fully functional PLC at hand. In that respect, inefficient loading of leader peptides in TAP-positive cells can be expected, whereas in cells devoid of TAP, this alternative ER entrance mechanism allows emergence of these peptides. So here, the PLC floats in the ER membrane and is dispatched from the entry site of peptides. Indeed, differential mass spectrometry analysis showed an enhanced presentation of leader peptides in cells lacking the peptide transporter (102). After all, MHC class I molecules get loaded with the available repertoire: from conventional or unconventional sources. But how do these “untapped” peptides find their way to empty MHC class I molecules at all? It has been shown that loading of peptides in MHC class I can occur with the minimal components of tapasin–ERp57–MHC class I complexes (103), but we have to assume that the chances of a peptide to find the PLC machinery in the ER are scarce. On the other hand, TAP-deficient cells harbor more peptide-receptive class I molecules compared to normal cells (104). These peptides might actually be actively chaperoned toward these open grooves. Chaperones with high peptide binding capacity in the ER are heat shock proteins (e.g., HSP96) and PDI, an isomerase that efficiently binds free peptide (105). It is possible that TAP-independent peptides are captured by these molecules and chaperoned to MHC class I. Recent studies suggest that the TAPBPR molecule (“Tapasin-like”) binds those MHC class I proteins that are not bound to tapasin in the PLC, so we might hypothesize that this pool of peptide-receptive grooves may function to load leader peptides (106). However, this still needs to be investigated.

Other ways to access peptide-receptive MHC has been proposed and are related to the intracellular traffic of hydrophobic peptides. Studies with several epitopes from the LMP2 protein of the EBV showed that peptides, which possess a high hydrophobicity index, were presented in a TAP-independent manner (107). The proposed mechanism describes the generation of these peptides outside the ER, since proteasome activity is needed for presentation, and subsequently free diffusion across the ER membrane possible due to their high hydrophobicity. These peptides might transgress membranes spontaneously or via alternative membrane transporters.

### Autophagy: MHC class i peptide loading in vesicular compartments

A recent study by Tey et al. showed that the processing of a peptide antigen from the human cytomegalovirus (HCMV) latency associated protein, pUL138, occurs entirely in the vesicular pathway and is mediated by autophagy (108). Also other examples of autophagy enhanced MHC class I presentation of viral antigens were reported (109, 110). During autophagy, large portions of cytoplasmic content, including proteins and organelles, are encapsulated in double membrane vesicles called autophagosomes (111–114). The autophagosomal membrane has been proposed to originate from the ER (115, 116) and peptide-receptive MHC class I molecules might be present in autophagosomes, allowing for loading of peptides in these vesicular compartments (117). In addition, recirculating MHC class I molecules from the cell membrane end up in endosomes and can have contact with autophagosomes before returning to the endocytic network. Transit for membrane-associated proteins between autophagosomes and endosomes has been observed by live cell imaging (118). Moreover, peptides generated by the proteasome seem to get access to the endocytic vesicular pathway as well. We recently found at least one example for this in our TEIPP repertoire that is generated by the proteasome but is TAP-independently presented (Table 1) (119). The presentation of this peptide was surprisingly enhanced by blockade of the proton pump in endosomes, indicating that this antigen most likely crosses the vesicular pathway (119). Finally, mass spectrometry analysis of the TAP-independent peptide repertoire pointed at proteins located in the vesicular compartment as an important source (120–122). Though these alternative processing and loading compartments have not fully been unraveled, these data strongly support the notion that the vesicular pathway and autophagy contributes to antigen presentation by MHC class I molecules.

## In Conclusion

**Figure 2:**
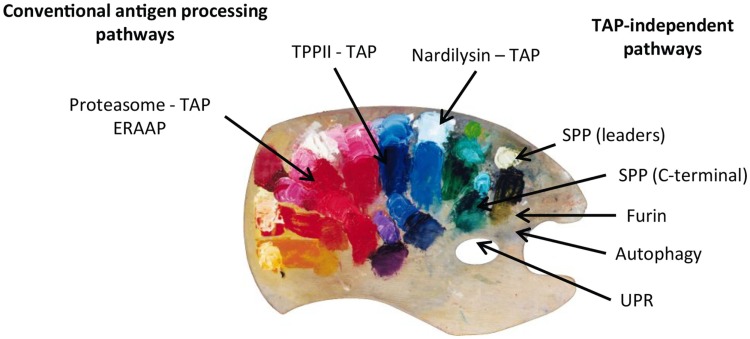
**Peptides presented on MHC class I molecules in normal and TAP-deficient cells derive from different MHC class I processing pathways**. Peptides derived from each pathway are combined in different proportions and combinations. The MHC class I peptide repertoire can therefore be compared to a painter’s palette where the different “colors” (peptides) are mixed and used to create a colorful and complex “picture.”

On summarizing, we can conclude that, even though the conventional proteasome-TAP pathway represents the major source of the MHC class I peptide repertoire, alternative processing pathways clearly complement the total pool. This multitude of MHC class I processing pathways can be compared to a colorful palette where the painter combines the different colors that will end up in different proportions and combinations in the final picture (Figure 2). In situations of viral infections, cellular transformation, or other initiators of stress, the contribution of these alternative pathways might gain importance. The SPP and maybe their family members are convincing examples of alternative proteolytic systems that feed the alternative routes of antigen presentation. The precise molecular identification of alternative loading mechanisms unto peptide-receptive MHC class I molecules, whether it be in the ER or in autophagosomes, still needs in-depth investigation. In that respect, peptides can walk on multiple different paths before ending up in the grooves of MHC class I and therefore illustrate the old expression that multiple roads lead to Rome.

## Conflict of Interest Statement

The authors declare that the research was conducted in the absence of any commercial or financial relationships that could be construed as a potential conflict of interest.
